# The emerging role of ferroptosis in non-cancer liver diseases: hype or increasing hope?

**DOI:** 10.1038/s41419-020-2732-5

**Published:** 2020-07-09

**Authors:** Lihong Mao, Tianming Zhao, Yan Song, Lin Lin, Xiaofei Fan, Binxin Cui, Hongjuan Feng, Xiaoyu Wang, Qingxiang Yu, Jie Zhang, Kui Jiang, Bangmao Wang, Chao Sun

**Affiliations:** 1 Department of Gastroenterology and Hepatology, Tianjin Medical University General Hospital, Anshan Road 154, Heping District, Tianjin, 300052 China; 2 Tianjin Institute of Digestive Disease, Tianjin Medical University General Hospital, Anshan Road 154, Heping District, Tianjin, 300052 China; 3 Tianjin Key Laboratory of Digestive Disease, Tianjin Medical University General Hospital, Anshan Road 154, Heping District, Tianjin, 300052 China; 4 Department of Gastroenterology, Tianjin Medical University General Hospital Airport Hospital, East Street 6, Tianjin Airport Economic Area, Tianjin, 300308 China; 5 Department of Nutriology, Tianjin Third Central Hospital, Jintang Road 83, Hedong District, Tianjin, 300170 China

**Keywords:** Cell death, Gastrointestinal diseases

## Abstract

Ferroptosis is an iron- and lipotoxicity-dependent form of regulated cell death (RCD). It is morphologically and biochemically distinct from characteristics of other cell death. This modality has been intensively investigated in recent years due to its involvement in a wide array of pathologies, including cancer, neurodegenerative diseases, and acute kidney injury. Dysregulation of ferroptosis has also been linked to various liver diseases and its modification may provide a hopeful and attractive therapeutic concept. Indeed, targeting ferroptosis may prevent the pathophysiological progression of several liver diseases, such as hemochromatosis, nonalcoholic steatohepatitis, and ethanol-induced liver injury. On the contrary, enhancing ferroptosis may promote sorafenib-induced ferroptosis and pave the way for combination therapy in hepatocellular carcinoma. Glutathione peroxidase 4 (GPx4) and system x_c_^−^ have been identified as key players to mediate ferroptosis pathway. More recently diverse signaling pathways have also been observed. The connection between ferroptosis and other forms of RCD is intricate and compelling, where discoveries in this field advance our understanding of cell survival and fate. In this review, we summarize the central molecular machinery of ferroptosis, describe the role of ferroptosis in non-cancer hepatic disease conditions and discuss the potential to manipulate ferroptosis as a therapeutic strategy.

## Facts

1. Ferroptosis execution is initiated with iron accumulation and overwhelming lipid peroxidation.
2. GPx4 and system x_c_^−^ are crucial ferroptosis pathway components.
3. Accumulating evidence has addressed that blockage of ferroptosis can mitigate the progression of liver damages of various etiologies.
4. Induction of ferroptosis may be beneficial for adjusting drug resistance against HCC.
5. Therapeutic strategy specific to hepatic disease on divergent cell type would be prioritized due to complicated function of ferroptosis itself.

## Open questions

1. Can we have unanimous modality for determining ferroptosis machinery in near future?
2. Could the results derived from experimental animals be smoothly translated to human pathophysiology?
3. What is the interplay between ferroptosis and other types of RCD?
4. Is it possible to target specific hepatocyte subtypes for regulating ferroptotic process in divergent disease context?

## Introduction

**Fig. 1:**
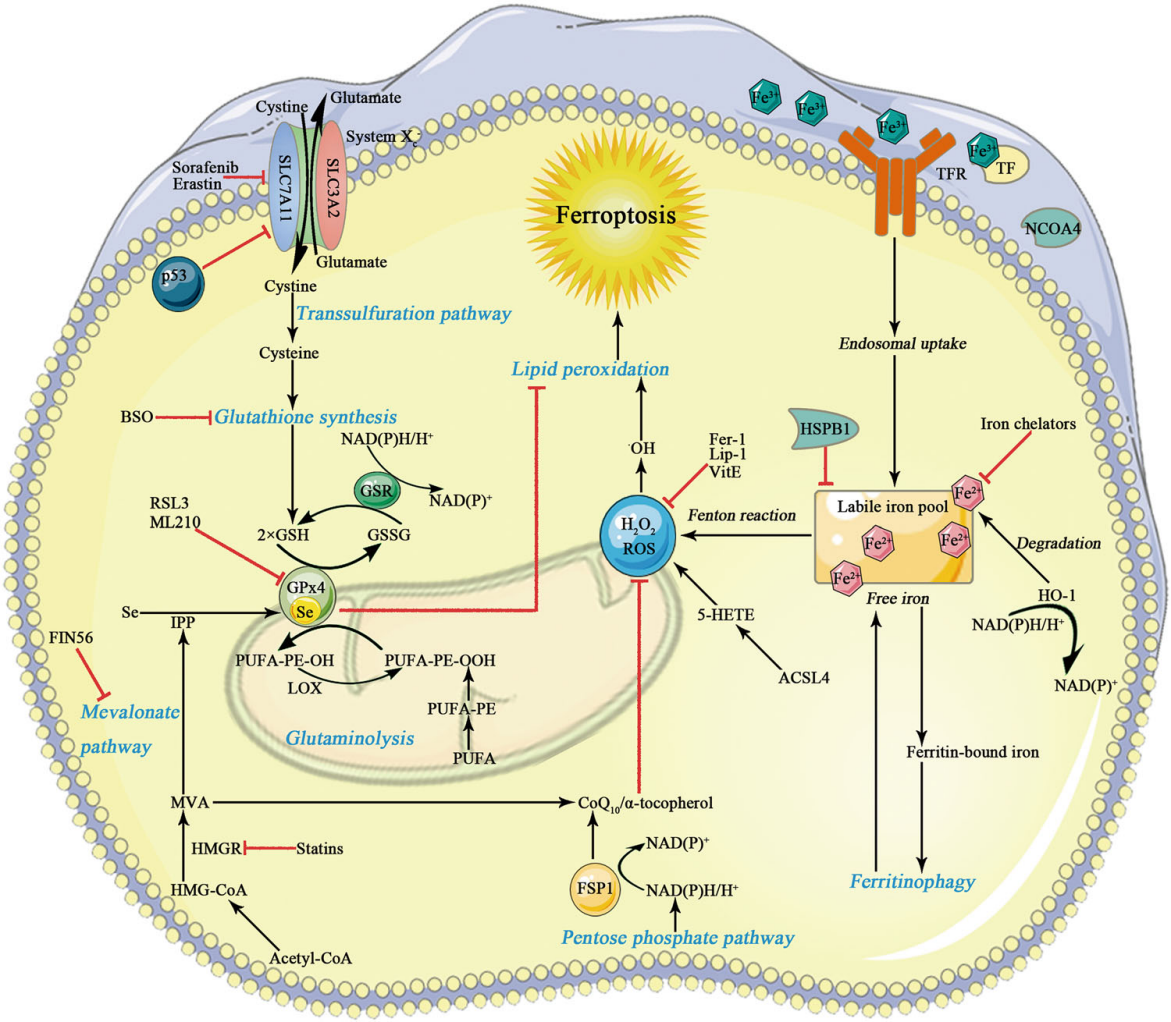
Metabolic pathways and key molecular mechanisms of ferroptosis. Initiation and execution of ferroptosis is attributed to accrued intracellular iron that is probably accentuated by ferritinophagy, disrupted mitochondrial function, a depletion of GSH that could result from deranged cysteine transport and GPx4 inactivation. The system x_c_^−^ (consisting of two subunits SLC7A11 and SLC3A2) is responsible for redox balance by uptake of extracellular cystine at the exchange of intracellular glutamate at 1:1 molar ratio. Inside the cell, cystine is reduced to cysteine by GSH and subsequently used for biogenesis of GSH. GPx4 is the core GSH utilizing enzyme, and it efficiently represses detrimental LOX overactivation and lipid peroxidation. Low GPx4 activities give rise to elevated accumulation of ROS and consequently to ferroptosis induction. A series of ferroptotic cell death inhibitors and activators have been well established in the past decades that interfere with different upstream events. GSH glutathione, GPx4 glutathione peroxidase 4, SLC7A11 solute carrier family 7 member 11, SLC3A2 solute carrier family 3 member 2, LOX lipoxygenases, ROS reactive oxygen species.

**Fig. 2:**
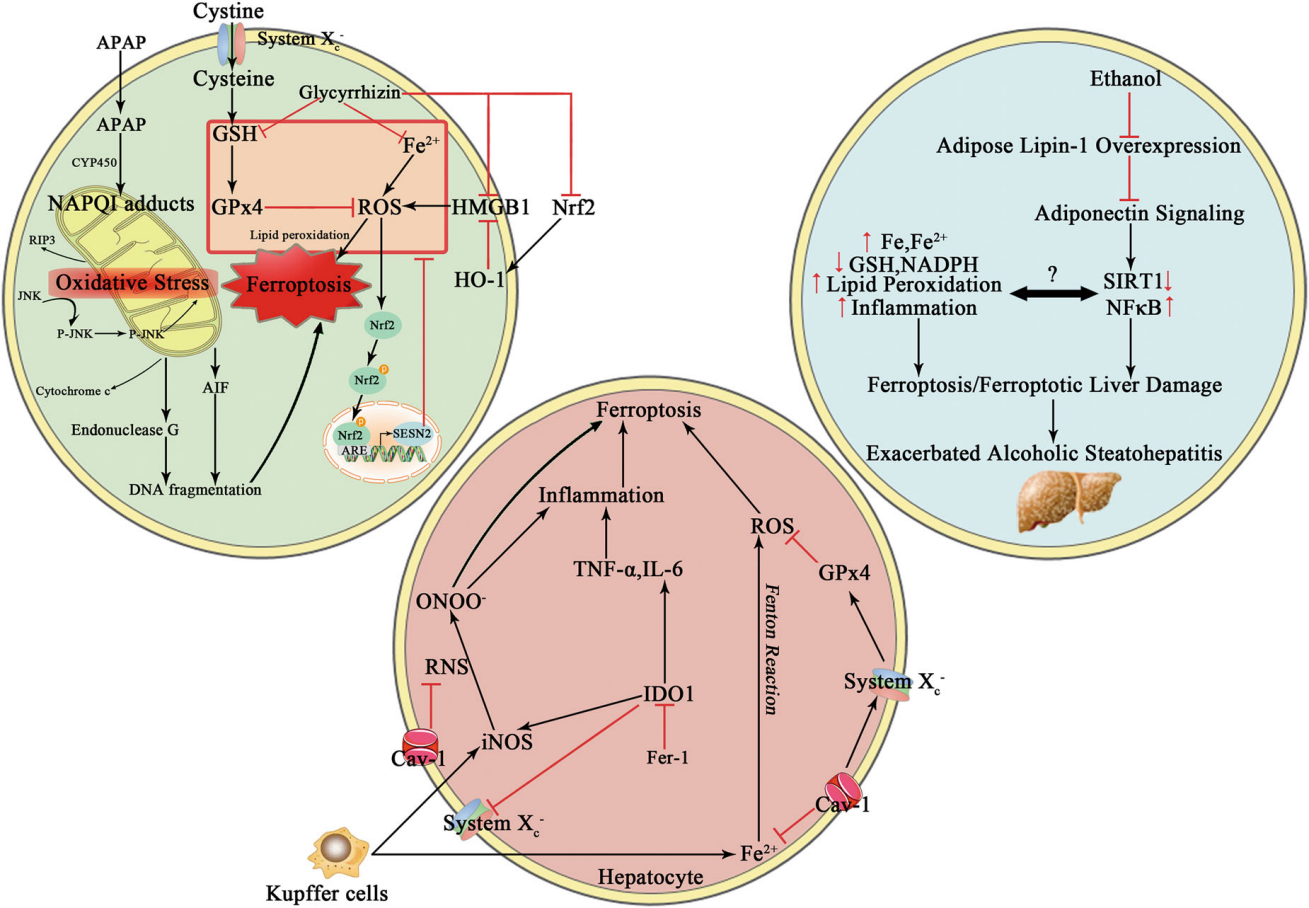
The function and possible mechanisms of ferroptosis in various liver injury. Dysregulation of ferroptosis has been linked to various liver diseases and its modification may provide a hopeful and attractive therapeutic prospect. Indeed, targeting ferroptosis may prevent the pathophysiological progression of several liver injuries, such as acute liver failure, alcoholic liver disease, NAFLD and immune-mediated hepatitis. The underpinning mechanisms include interplay between antiferroptotic action and other bioactivities including anti-inflammatory, antioxidant action and regulation of immunogenic response. APAP, acetaminophen, GSH, glutathione, GPx4, glutathione peroxidase 4, ROS, reactive oxygen species, HMGB1, high mobility group protein B1, HO-1, heme oxygenase-1, Nrf2, nuclear factor erythroid 2-related factor 2, SIRT1, Sirtuin1, IDO1, indoleamine 2,3-dioxygenase 1, iNOS, inducible nitric oxide synthase, RNS, reactive nitrogen species, Cav-1, Caveolin-1.

Ferroptosis is a newly identified type of regulated cell death (RCD). Morphologically, ferroptosis is characterized by smaller mitochondria with condensed, ruptured outer membrane as well as vestigial crista^1,2^. Biochemically, ferroptosis execution is initiated with iron accumulation, excessive reactive oxygen species (ROS) production and overwhelming lipid peroxidation^3^. Excess iron mediates ferroptotic process by generating lethal ROS via Fenton action. In this manner, circulating iron appears in the form of ferric iron (Fe3+), which binds to transferrin. The transferrin receptor 1 imports Fe3+ through cellular membrane and then locates in the endosome, where Fe3+ is subsequently reduced to ferrous iron (Fe2+). Released Fe2+ from the endosome is transported into a labile iron pool (LIP) in the cytosol. Moreover, excessive iron is stored in ferritin, a protein complex characterized by two subunits comprising ferritin light chain and ferritin heavy chain 1. These implicate that reduced iron storage and increased iron intake may facilitate iron overload in ferroptosis. Of note, iron chelator (e.g., deferoxamine) can efficiently eliminate iron overload thus inhibits erastin-induced ferroptosis, whereas exogenous iron supplement aggravates erastin-mediated cell death^4^. This modality is also proposed to be interconnected with other forms of RCD through preferential release of damaging molecules, leading to tissue injury and organ dysfunction^5^. Glutathione peroxidase 4 (GPx4) and system x_c_^−^ are crucial ferroptosis pathway components (Fig. 1). The system x_c_^−^/GPx4 axis favors the cellular import of cystine, chronological reduction to cysteine and biogenesis of glutathione (GSH). As a result, ferroptotic cell death can be induced by two routes, that is, by either inactivating activities of GPx4 or by aggravating the LIP. In a word, the cellular systems orchestrate the uptake and utility of iron, which are indispensable for the induction of ferroptosis. In addition to iron-mediated ROS generation by Fenton chemistry, GSH depletion and overwhelming lipid peroxidation are also required to the induction and execution of ferroptotic process. ROS react with the polyunsaturated fatty acids (PUFAs) of lipid membranes. Oxidation of PUFAs by lipoxygenases (LOX) gives rise to the accumulation of peroxides responsible for the production of lipid peroxide breakdown compounds. Another critical participator in the ferroptotic cell death process, that is, acyl-CoA synthetase long-chain family member 4 (ACSL4) builds on its capability to ligate coenzyme A to long-chain PUFAs, which can then esterified into membrane phospholipids and oxidized to transmit the ferroptosis signal (see discussion below)^6^. In case of being seeded into membrane, these PUFAs undergo peroxidation and drive massive lipid peroxidation and ferroptosis execution^7^. Erastin can inhibit the uptake of cystine, subsequently resulting in GSH depletion and GPx4 inactivation. RAS-selective lethal 3 (RSL3), retaining enzymatic active site, binds to the selenocysteine of GPx4 and exerts inducible effect. On the contrary, commonly used ferroptosis inhibitors are believed to eliminate lipid radicals such as ferrostatin-1 (Fer-1) and liproxstatin-1 (Lip-1)^2,8^. Dysregulation of ferroptosis is observed in a wide range of pathological conditions, including chronic pulmonary obstructive disease, intracerebral hemorrhage, degenerative diseases, acute kidney injury, and cancer^2,9–12^. Accumulating evidence has addressed that blockage of ferroptosis can mitigate the development and progression of a number of liver diseases, including hemochromatosis, immune-mediated hepatitis, alcoholic steatohepatitis, and acute liver failure (Fig.2 and Table 1)^13–16^. Nonetheless, in some circumstance, induction of ferroptosis may be beneficial for adjusting drug resistance and contributing to combined treatment regimens against hepatocellular carcinoma (HCC)^17–19^. Likewise, targeting specific hepatocyte subtypes must be considered, such as hepatic stellate cell (HSC) in the fibrogenic processes, since RCD might display pleiotropic role dependent on the cell type and different disease context (Table 2)^20^. Collectively, we suppose that therapeutic strategy specific to hepatic disease on divergent cell type would be prioritized due to complicated function of ferroptosis itself. In current review, we focus on the relevance of ferroptosis and pathophysiological aspects of ferroptotic manipulation in a variety of non-cancer liver diseases, whereas the role of ferroptosis in HCC has been well documented by others and ours^21,22^.

**Table 1:** The function and mechanisms of ferroptosis in various liver injury.

Disease	Ref.	Model	Compound/target	Effect	Mechanism/phenotype
Acute liver failure	^16^	LPS/GalN-induced mice L02 cells	Glycyrrhizin	Inhibition of ferroptosis	NRF2, HO-1, and GPx4↑ HMGB1↓
	^58^	LPS/GalN-induced mice	Promethazine	Inhibition of ferroptosis	NA
Acute liver injury	^60^	PHZ-induced mice/Ad-Sesn2 infected mice HepG2 cells	Sestrin2	Inhibition of ferroptosis	NRF2, TFR1, ferroportin↑
Alcoholic liver disease	^15^	SIRT1iKO mice	Intestinal sirtuin1 (deficiency)	Inhibition of ferroptosis	Pro-inflammatory molecules LCN2, SAA1↓ Redox active iron–sulfur CISD1/2↓
	^65^	*Lpin1*-Tg mice	Adipose-specific lipin-1 (overexpression)	Induction of ferroptosis	Adiponectin-sirtuin1, adiponectin-FGF15 axis↓ NF-κB↑
NAFLD	^74^	CDE diet/MLKL^−/−^ mice	Trolox/DFO	Inhibition of ferroptosis	TNF-α, IL-1β, IL-6↓
Immune-mediated hepatitis	^14^	ConA-induced/Cav-1^−/−^mice	Caveolin-1	Inhibition of ferroptosis	RNS, iNOS↓
	^83^	ConA-induced/IDO1^−/−^mice	IDO1 (deficiency)	Inhibition of ferroptosis	xCT↑ RNS↓
Ischemia/reperfusion injury	^100^	I/R mice	Liproxstatin-1	Inhibition of ferroptosis	MPO↓
	^104^	HID-fed I/R mice	Ferrostatin-1/DFO/α-Tocopherol	Inhibition of ferroptosis	PTGS2↓ Inflammatory cytokines, Ly6G/Mac2↓

*LPS* lipopolysaccharide, *GalN* d-galactosamine, *NRF2* nuclear factor erythroid 2-related factor 2, *HO-1* heme oxygenase-1, *GPx4* glutathione peroxidase 4, *HMGB1* high mobility group protein B1, *TFR1* transferrin receptor 1, *LCN2* lipocalin 2, *SAA1* serum amyloid A1, *CISD* CDGSH iron sulfur domain, *NAFLD* nonalcoholic fatty liver disease, *CDE* choline-deficient ethionine-supplemented, *MLKL* mixed lineage kinase domain-like protein, *DFO* deferoxamine, *ConA* concanavalin A, *Cav-1* caveolin-1, *RNS* reactive nitrogen species, *iNOS* inducible nitric oxide synthase, *IDO1* indoleamine 2,3-dioxygenase 1, *MPO* myeloperoxidase, *HID* high iron diet, *PTGS2* prostaglandin-endoperoxide synthase 2, *NA* not applicable.

**Table 2:** The function and mechanisms of ferroptosis in fibrosis.

Model	Ref.	Compound/target	Effect	Fibrogenic marker	Mechanism
CCl_4_-induced fibrosis	^93^	MgIG	Induction of HSC ferroptosis	α-SMA, collagen1, fibronectin, desmin	TGF-βR1, PDGF-βR HO-1↑
CCl_4_-induced fibrosis	^94^	Artemether	Induction of HSC ferroptosis	α-SMA, collagen, fibronectin	PDGF-βR, EGFR↓p53↑
BDL-treated fibrosis	^95^	ELAVL1	Activation of HSC ferritinophagy/ferroptosis	ACTA2, COL1A1	BECN1 mRNA stability↓
BDL-treated fibrosis	^96^	ZFP36	Inhibition of HSC autophagy/ferroptosis	ACTA2, COL1A1	ATG16L1 mRNA decay↓
CCl_4_-induced fibrosis	^97^	Artesunate	Activation of HSC ferritinophagy/ferroptosis	α-SMA, collagen1, fibronectin	LC3-II↑ p62, FTH1, NCOA4↓

*CCl*_*4*_ carbon tetrachloride, *MgIG* magnesium isoglycyrrhizinate, *HSC* hepatic stellate cell, *α-SMA* alpha-smooth muscle actin, *HO-1* heme oxygenase-1, *BDL* bile duct ligation, *ELAVL1* ELAV like RNA-binding protein 1, *COL1A1* collagen type I alpha 1, *BECN1* beclin 1, *FN1* fibronectin 1, *NCOA4* nuclear receptor coactivator 4, *LC3* microtubule-associated protein light chain 3, *FTH1* ferritin heavy chain.

## Key mediators of ferroptosis machinery

### System x_c_−

System x_c_− is comprised of a functional subunit solute carrier family 7 member 11 (SLC7A11) and a regulatory subunit solute carrier family 3 member 2 (SLC3A2). This highly specific complex is responsible for redox balance by uptake of extracellular cystine (the oxidized form of cysteine) at the exchange of intracellular glutamate at a 1:1 molar ratio^23^. After imported by system x_c_^−^ across the plasma membrane, cystine is reduced to cysteine by GSH and subsequently used for biosynthesis of GSH. The functioning performance of GSH, as a prevailing endogenous antioxidant, relies on the availability of cysteine, the bioactivity of glutamate–cysteine ligase and the sulfur amino acid precursor. In addition, cysteine represents the rate-limiting substrate in synthesis of GSH. Taken together, any approach dampening the levels of intracellular cysteine and consequent deprivation of GSH contents potentiates ferroptosis. SLC7A11 is subject to complicated transcriptional regulation^24^. The expression of SLC7A11 can de elicited by oxidative and amino acid depletion in an activating transcription factor 4 (ATF4)-dependent and nuclear factor erythroid 2-related factor 2 (NRF2)-dependent manner. Accordingly, tumor growth can be remarkably inhibited in a number of cell lines bearing *SLC7A11* knockout^25,26^. Erastin is capable of reducing GSH level by repressing system x_c_^−^ activity and activating the endoplasmic reticulum (ER) stress response, favoring ROS accumulation in ferroptotic process^27^. Moreover, p53 has been proved to downregulate system x_c_^−^ expression resulting in cystine starvation and susceptibility to ferroptosis, thereby probably being beneficial for cancer eradication^28,29^.

### GPx4

GPx4 was firstly identified in 1982 as the second mammalian GSH peroxidase, then documented to be the core upstream regulator of ferroptosis in 2014^30,31^. It is capable of reducing phospholipid hydroperoxides and cholesterol hydroperoxides to their counterparts, thus interfering with the lipid peroxidation chain reaction^32^. GPx4 absence through conditional depletion gives rise to non-apoptotic cell death on account of massive lipid oxidation^33^. GPx4 is indispensable for preserving tissue homeostasis and avoiding cell death in multiple organ/tissue damage^34^. Emerging evidence has addressed that genetic deletion of GPX4 can induce ferroptosis in an iron-, MEK-, and ROS-dependent manner^35^. RSL3 embraces an electrophilic chloroacetyl moiety, as the first depicted GPx4 inhibitor, covalently reacting with the nucleophilic active site Sec of GPx4, then resulting in irreversible inactivation of this enzyme^31,36^. Lipoxygenases (LOX) have been suggested to trigger ferroptotic cell death by inducing peroxides in fatty acid residues of phospholipids. In accordance, compounds that increase LOX expression may retain higher cellular hydroperoxide levels predisposing cells to ferroptosis^37^. It is evident that GPx4 counteracts the effect of peculiar LOX by controlling the cellular peroxide tone^38^. On the contrary, in *GPx4* knockout-induced cells representing ferroptosis features, a phenotypic screening campaign identified that Lip-1 as the first efficacious ferroptosis inhibitor in vivo^2^. Administration of Lip-1 profoundly improved survival in a genetic model of acute renal failure and mitigated hepatic ischemia-reperfusion (I/R) injury in mice. Collectively, all above observations confirmed GPx4 as a negative regulator of ferroptosis machinery.

### ACSL4

By performing genetic screens in disparate cell lines, it has been determined that ACSL4 as a pivotal downstream player in the ferroptotic process^6,39^. ACSL family is consisted of ACSL1, ACSL3, ACSL4, ACSL5, and ACSL6, all of which are expressed at the mitochondrial and ER outer membrane. ACSL4 is involved in the process of ferroptosis because of its ability to ligate coenzyme A to long-chain PUFAs. In case of impaired functionality of GPx4, these PUFAs located in cellular membrane, may undergo peroxidation, trigger excess lipid peroxidation and ferroptosis^7^. In a conditional ACSL4 knockout model, the lipid peroxides and ferroptosis were profoundly inhibited^6^. The reversal was observed by transgenic overexpression of ACSL4 in cells, meanwhile GPx4 bioactivity was selectively suppressed to prevent confounding effect. Moreover, the knockdown of ACSL4 confers cells to ferroptosis resistance, whereas ACSL4 overexpression restores erastin-induced ferroptosis sensitivity^40^. Taken together, these studies imply that ACSL4 contributes to ferroptosis execution.

### NRF2

NRF2 is regarded as a mainstay for modulating antioxidant response, as a myriad of its downstream targeting genes attributable to maintaining redox balance in cells^41^. Under normal conditions, protein levels of NRF2 are basally retained by Kelch-like ECH-associated protein 1. In response to various stressors, NRF2 can translocate to the nucleus to initiate the transcription of its targets, that is, antioxidant response element (ARE)-containing genes. Furthermore, two crucial targets whose inhibition initiates ferroptosis, SLC7A11 and GPx4, are well documented to be regulated by NRF2. Of note, trigonelline has been found to enhance sorafenib-induced and artesunate-induced ferroptotic cell death in divergent cell lines^42,43^. The NRF2 expression level seems to correlate with ferroptosis sensitivity, as cancer cells with downregulated NRF2 are prone to pro-ferroptotic pharmaceuticals, whereas increased NRF2 expression prevents the initiation and execution of ferroptosis^44^. These observations implicate inhibitors of NRF2, as well as its downstream targets, could serve as powerful approaches to elicit ferroptosis-dependent cancer cell death.

### p53–p21

The p53 tumor suppressor protein is believed to repress carcinogenesis via apoptosis, cell cycle arrest as well as senescence. However, p53 retains its antitumor activity in specific mutations variant (i.e., p53-KR), suggesting other biological pathways are possibly involved. Recent study indicated p53-KR interrupts with the import of cystine, and eventually results in GSH depletion and ferroptotic cell death^45^. By contrast, overexpression of wild-type p53 consistently inhibits ferroptosis by repressing the lipotoxicity due to ROS accumulation and improving the conservation of the cysteine-derived antioxidant GSH^46^. These findings raise possibility that p53–p21 pathway may aid in recycling GSH, decreasing the export or consumption of cellular GSH^47^. On the other hand, p21 is also known to mediate cell survival, metabolism, and oxidative stress in a p53-independent manner^48^.

## Ferroptosis in non-cancer liver diseases

### Acute liver injury/failure

Soon after the discovery of ferroptosis, several studies investigated whether this pathway is involved in experimental models of acute liver injury/failure. The most clinically relevant cause for drug hepatotoxicity and acute liver failure is intoxication of acetaminophen (APAP). The mechanisms of APAP-induced cell death have been immensely explored due to the availability of a mouse model that is close to human pathophysiology^49,50^. It has been reported that APAP overdose leads to dramatical decrease in intracellular GSH levels, a critical mediator for the activation of ferroptosis^51^. Moreover, the role of iron accumulation and lipid peroxidation has also been long identified as underpinning mechanisms of APAP-induced liver injury, but their contribution to ferroptotic cell death related to APAP is still enigmatic. Schnellmann et al. found the chelation of intracellular iron by DFO mitigates APAP-induced liver injury^52^. Fer-1 treatment is noted to promote cell viability in the case of APAP-treated primary mouse hepatocytes, implicating the presence of ferroptotic cell death. Sterile inflammation may also be involved in APAP-induced hepatotoxicity through inflammasome-dependent IL-1β release^53^. Intriguingly, it is well exploited that mitochondrial damage may result in inflammasome activation^54^. Emerging evidence implicates that mitochondria are more likely to play a crucial role in the progression of ferroptosis^55^. Whether targeting the mitochondrial metabolic and redox reaction with subsequent intervention on ferroptotic cell death may prevent this excessive inflammasome activation remains to be elucidated.

Acute liver failure is a rare, unexpected and severe consequence of abrupt hepatocyte damage, and has a rapid onset with a lethal outcome^56^. It is mainly caused by virus and autoimmune hepatitis (AIH), hepatic ischemia, drug-induced liver injury due to prescription drugs, and herbal as well as dietary supplements^56^. Oxidative stress may underline the pathophysiological correlation between acute liver failure and ferroptosis, as the accumulation of ROS culminates in ferroptosis execution^57^. In lipopolysaccharide (LPS) and d-galactosamine (GalN)-induced ALF mice, the protein levels of GPx4, NRF2, and heme oxygenase-1 (HO-1) were significantly decreased, whereas the level of high mobility group protein B1 (HMGB1) was increased^16^. Moreover, the levels of LDH, Fe^2+^, malondialdehyde (MDA) and ROS were increased, while the level of GSH was decreased. Treatment with glycyrrhizin, a HMGB1 inhibitor, could alleviate the degree of liver damage by targeting ferroptosis via inhibition of oxidative stress. Another state-of-the-art study aimed to screen cytochrome P450 substrate compounds with antiferroptotic bioactivity^58^. Their results showed promethazine can ameliorate LPS/GalN-triggered acute liver failure via suppression of lipid peroxidation and decreased cell death. The antiferroptotic action of promethazine is closely associated with the scavenging of lipid peroxyl radicals. Sestrin2 (Sesn2) responds to various stress, as a conserved antioxidant protein, and acts to restore homeostasis^59^. In mice pretreated with phenylhydrazine, a well-known iron overload liver injury model, injection of adenoviral Sesn2 completely abolished the elevated serum ALT/AST levels and altered histological changes^60^. Cells expressing Sesn2 were resistant to erastin-induced ferroptotic cell death, ROS formation or GSH depletion.

### Alcoholic liver disease

Alcoholic liver disease comprises a broad spectrum of detrimental conditions including steatosis, steatohepatitis, hepatitis, fibrosis/cirrhosis, to liver failure and HCC^61^. Ethanol results in enormous products of highly actively acetaldehyde, fatty acid ethyl esters, phosphatidylethanol, and ROS. These metabolites can hinder a number of cellular events such as proteostasis, lipogenesis, redox balance, and mitochondrial respiration, resulting in hepatocyte cell death^62^. Furthermore, long-term ethanol consumption leads to hepatomegaly and hepatic protein accumulation. Sirtuin1 (SIRT1), a class III histone deacetylase, serves as a protective player against alcoholic steatohepatitis in rodents and humans^63,64^. Flox control (i.e. WT) mice presented with more severe liver injury and hepatic inflammation, fed with a chronic-plus-binge ethanol administration, compared with mice bearing intestinal specific SIRT1 deletion (SIRT1iKO)^15^. The protective effect of intestinal SIRT1 deficiency may be attributable to alleviating iron metabolism dysfunction, elevating GSH contents and attenuating lipid peroxidation. Moreover, a panel of genes implicated in ferroptosis process was normalized in the livers of ethanol-fed SIRT1iKO mice. Zhou et al. constructed experimental alcoholic steatohepatitis by pair-feeding ethanol to adipose-specific lipin-1 overexpression transgenic (*Lpin1*-Tg) mice and WT mice^65^. *Lpin1*-Tg mice exhibited deleterious steatosis, augmented inflammation, aggressive hepatobiliary injury and fibrogenic responses. The exacerbated steatohepatitis in ethanol-fed *Lpin1*-Tg mice was associated with massive iron accumulation, abnormal iron distribution, decreased GSH, increased MDA levels, and impaired ferroptosis-related gene expression, all of which indicated the pivotal role of hepatic ferroptosis. Notably, GPx4, the core regulator of ferroptosis execution, was not significantly changed in ethanol challenged mice model at both mRNA gene and protein expression levels. Thus we can infer that alternate pathway such as depleting cystine and suppressing glutamate antiporter system x_c_^−^ (xCT) may sensitize liver to ethanol-inducible ferroptosis.

### Nonalcoholic fatty liver disease

Nonalcoholic fatty liver disease (NAFLD) now is the leading cause of chronic liver diseases and HCC worldwide, along with the rising incidence of obesity and diabetes^66^. NAFLD is a generic designation comprising nonalcoholic fatty liver and nonalcoholic steatohepatitis (NASH). NAFLD is characterized by cellular accumulation of lipid droplet, hepatocyte cell death, infiltration of immune/inflammatory cells and fibrosis to some extent. In some individuals simple steatosis proceeds to NASH, which is a risk factor for cirrhosis and tumorigenesis^67^—4–27% of patients with cirrhotic NASH develop HCC^68^. Some studies have demonstrated that lipotoxicity, such as oxidative stress and insulin resistance, is prompted by excessive triglycerides and free fatty acids in NAFLD^69,70^. The oxidative stress are aberrantly marked by MDA and 4-hydroxinonenal (4-HNE), secondary products of lipid peroxidation, in NASH subjects^71^. In addition, the iron accumulation arising from metabolic aberration may aggravate the process of NASH, since some NASH patients manifest hepatic siderosis while removal of iron reversed liver damage^72,73^. Collectively, there is evidence supporting the involvement of ferroptosis in NASH as described above. Most recently, in a choline-deficient, ethionine-supplemented (CDE) diet model, Tsurusai et al. revealed that ferroptosis precedes other type of cell death, thus giving cues to initiate inflammation in steatohepatitis^74^. Trolox and DFO, two ferroptosis inhibitors, repressed ferroptotic cell death, infiltration of immune cells and inflammatory cytokines expression (TNF-α/IL-1β/IL-6) in the liver of CDE-fed compared with normal diet-fed mice. By contrast, the initial cell death could not be blocked by Nec-1 (specific necroptosis inhibitor) or by using mixed lineage kinase domain-like protein (MLKL) knockout mice. Taken together, it is likely to take ferroptosis into account for the therapeutic strategy of NASH. However, further experimental validation using core ferroptosis-related gene knockout mice and featuring more phenotypic observation (mitochondrial atrophy/lipid peroxidation) is inevitably needed on NASH model.

### Immune-mediated hepatitis

AIH is a persistent and relapsing immune-mediated liver injury, which is characterized by chronic hepatitis of varying severity^75^. AIH embraces a significant risk of developing into end-stage liver disease (cirrhosis and liver failure) if without timely and effective therapy. The etiology of AIH is complicated, probably including environmental, genetic, and epigenetic drivers of inflammation. AIH is pathologically characterized by inflammatory infiltration of lymphocytes, plasma cells, and eosinophils in the liver, however the detailed mechanism of hepatocyte cell death regulation is still elusive^76^. Concanavalin A (ConA) stimulation can lead to fulminant immunological liver injury, thus mimic clinical features of immunogenic hepatitis to investigate the mechanisms and therapy of AIH^77^. Notably, ConA-induced liver injury could not be mitigated by pretreatment with apoptosis inhibitor, rendering this model applicable for exploiting precise role of necrotic cell death of hepatocytes in AIH^78^. It is currently accepted that excessive ROS and reactive nitrogen species (RNS) contribute to oxidative tissue damages and dampen cell survival^79,80^. In a Caveolin-1 (Cav-1) deficient ConA-triggered mice model, hepatocellular death and ferroptosis have been more pronounced with significant nitrogen stress^14^. Depleting Cav-1 expression may be attributable to the inducible nitric oxide synthase (iNOS) accumulation produced by Kuffer cells, and further positively impacts ferroptosis development in AIH. Moreover, Fer-1 could protect against ConA-induced hepatitis relevant to Cav-1 reversion and RNS suppression. Indoleamine 2,3-dioxygenase 1 (IDO1) is an intracellular heme enzyme, which serves as an important immune regulator and is related to the production of Fe^2+^ ^81,82^. Intervention of IDO1 and the administration of ferroptosis inhibitor mitigated ferroptotic cell death and nitrative stress in the ConA-challenged mice^83^. The underpinning mechanism of IDO1-dependent ferroptosis comprises its role of system x_c_^−^ modulator as well as versatile inflammatory state modifier. Finally, these data suggest that ferroptosis may play a part in immune-mediated hepatitis, and targeting this modality is a promising therapy in AIH considering the side effects of corticosteroids and other immunosuppressive agents^84^.

### Fibrosis

As well documented, the cornerstone in the fibrogenic process is the activation of HSC. When driven by several liver damage elements, quiescent HSC transdifferentiate into contractile myofibroblast capable of matrix production, subsequently resulting in fibrosis^85,86^. It has been widely addressed that the inhibition of HSC proliferation and induction of several types of cell death, including apoptosis, autophagy, senescence and pyroptosis are effective to treat hepatic fibrosis^87–90^. Recent studies have found that modulation of ferroptosis might serve as anti-fibrotic therapy (Fig. 2 and Table 2).

HO-1 rapidly responds to various stimuli, and acts as a gatekeeper in multiple pathological states by regulating ferroptosis^91,92^. Sui et al. found that HO-1 overexpression mediates magnesium isoglycyrrhizinate-induced anti-fibrotic effect by inhibition of HSC proliferation and activation^93^. The dependency for HO-1 in HSC ferroptosis arises from not only expression of HO-1, but also its abundance in nucleus as well as alteration of HO-1 downstream factors (transferrin/transferrin receptor/ferritin heavy chain). A more recent study revealed that artemether (ART) promoted the expression and nuclear import of p53, facilitating inhibition of profibrotic performance of activated HSC by correcting iron metabolism and lipid peroxides. On the contrary, inhibition of ferroptosis by Fer-1 completely abolished ART-induced antifibrosis effect^94^. The same research group further corroborated that manipulation of ferritinophagy is required for RNA-binding protein (ELAVL1/HuR or ZFP36) mediated ferroptotic bioactivity on HSC, conferring therapeutic target for the treatment of liver fibrosis. Notably, the upregulation of ELAVL1 (by abrogating *Beclin* mRNA stability) and downregulation of ZFP36 (by reversing ATG16L1 mRNA decay) could enhance sorafenib-elicited HSC ferroptosis for ameliorating murine liver fibrosis^95,96^. It has also been suggested that ferritinophagy-mediated HSC ferroptosis was responsible for artesunate-induced antifibrosis efficacy^97^. Altogether, the current state of the literature provides new clues for further pharmacological study via interference with autophagy-ferroptosis interplay on HSC.

### Other liver diseases

Hereditary hemochromatosis (HH) is an iron-overload disease due to mutations in genes whose protein products limit iron absorption^98^. In HH, iron accumulates in various organs and generates ROS, subsequently leading to cell death, oxidative injury and severe complications^99^. Wang et al. found that iron overload could trigger ferroptosis both in vivo and in vitro^13^. In addition, using *SLC7A11* knockout mice, they concluded that the absence of SLC7A11 facilitates iron overload-elicited ferroptosis on account of impaired cystine uptake and increased ROS production. At last, this iron metabolism-related ferroptosis seems independent of ER stress, MAPK signaling pathway or autophagy.

A growing body of literature has suggested that ferroptosis may be involved in the pathogenesis of I/R injury in several organs^2,100–102^. Moreover, hepatic I/R injury induces inflammatory cascade and immunological reactions, which dampens normal graft liver function and leads to poor prognosis of the recipients^103^. Iron load of the donor has been proved to be an independent risk factor for hepatic I/R injury in pediatric living donor LTx^104^. In a murine model of hepatic I/R injury, lipid peroxidation and ferroptosis marker *PTGS2* were elicited, all of which were markedly prevented by Fer-1 or α-tocopherol. The mice fed with high iron diet represented deleterious I/R injury due to overload, and these manifestation was attenuated by DFO^104^. Furthermore, Li et al. found that ferroptosis inhibition by Lip-1 significantly decreased myeloperoxidase (MPO) activity and mitigated histological injury in liver^100^.

Malaria comprises a significant health burden upon the world, with over 200 million cases occurring every year. The vast majority of morbidity and mortality from malaria is estimated to be 194 million cases and 619 000 deaths each year^105^. The clinical phenotypes ranges from multi-organ complications, severe anemia to cerebral malaria including coma and death^106^. Plasmodium parasites are causative agents of malaria and maintained between Anopheles mosquitoes and mammalian hosts in a complex life cycle^107^. They grow and develop in a broad range of host environments, where diversity is testament to their exceptional adaptability and gives rise to a challenge for developing strategies to reduce the disease burden and transmission^108^. After transmission to mammalian hosts, *Plasmodium* parasites travel through the vessels to the liver, where each parasite infects a hepatocyte to form a liver stage (LS) parasite^109^. A recent study showed that hepatocellular SLC7A11-GPx4 signaling pathway modulates *Plasmodium* infection through the generation of ROS and lipid peroxidation^110^. Targeting p53 in curtailing malaria LS infection depends on ROS production and lipid peroxidation, whereas noncanonical effect of p53 are also crucial for LS infection.

## Conclusions and future research perspectives

**Fig. 3:**
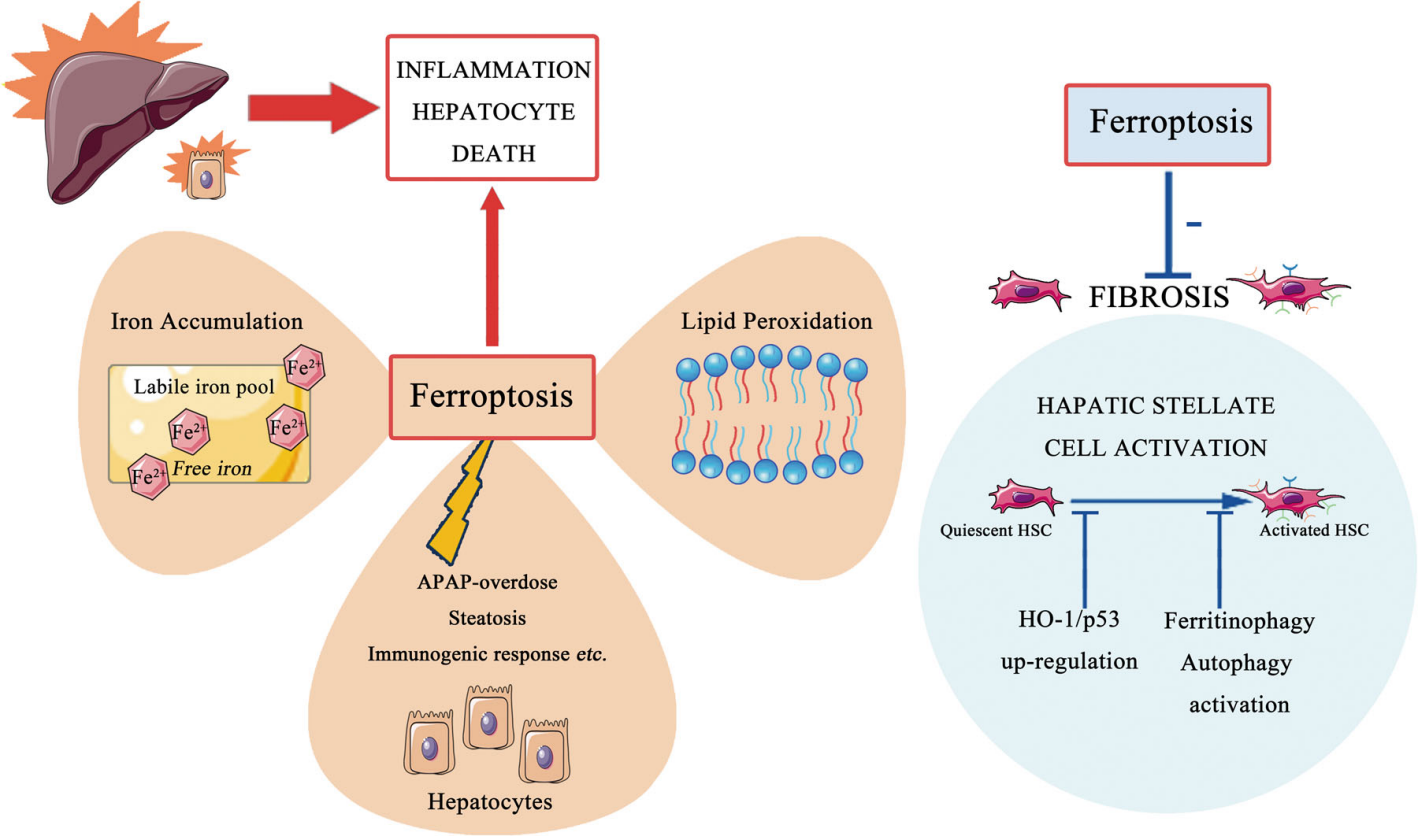
The dual role of ferroptosis in distinct hepatic entities. The cornerstone in the fibrogenic process is the activation of HSC. When driven by hepatic damage elements, quiescent HSC transdifferentiate into contractile myofibroblast capable of matrix production, subsequently resulting in fibrosis. Recent studies have found that modulation of ferroptosis might serve as anti-fibrotic therapy. Moreover, ferritinophagy-mediated HSC ferroptosis was also responsible for anti-fibrosis efficacy. HSC, hepatic stellate cell, APAP, acetaminophen, HO-1, heme oxygenase-1.

Ferroptosis is a newly identified form of cell death which might be involved in the onset and development of a variety of pathogenic conditions. The extensive research progression in ferroptosis has advanced our current understanding with respective to cell death cascade. Furthermore, the precise role of ferroptosis seems to be cell type-dependent and disease context-dependent (Fig. 3), whereas it is still to be clarified whether the results derived from experimental animals could be smoothly translated to human pathophysiology. Intriguingly, some studies provide an additional impetus to scrutinize the connection between ferroptosis and other constituents of RCD such as apoptosis and autophagy/ferritinophagy. It is plausible to selectively modulate a specific cellular death identity for eliminating and/or preventing the damaged tissue from devastating events, consequently maintaining intrinsic homeostasis in a complicated multi-organ system. However, there are some outstanding issues which should be stated in future studies.

First, it should be noteworthy that currently available data are mostly obtained from murine models of diverse hepatic diseases or from in vitro studies. These results should be interpreted with caution, since we are now lacking in unanimous modality for determining ferroptosis machinery. According to established characteristics of ferroptosis, it is common to evaluate ferroptotic cell death by cellular necrotic staining like TUNEL assay^4^. Meanwhile, we can determine the levels of Fe^2+^, MDA, 4-HNE, and BODIPY 581/591 C11 to estimate iron-related lipid peroxidation^9,111^. Furthermore, the GSH/GSSG and GPx4 expression levels should be assessed to examine the suppression of antioxidants during ferroptosis^112^. At last, it is evident to observe condensed mitochondrial membrane with smaller volume, in addition to the reduction or vanishing of mitochondria crista and ruptured outer membrane^55^. It is necessary to integrally conduct ferroptotic assays from distinct aspects for pinpointing the occurrence of this modality. Likewise, this would be greatly assisted and urgently needed to discover feasible biomarkers and approaches that affording comprehensive characterization of ferroptosis.

Second, the application of ferroptosis inhibitor like Fer-1 by the majority of publications seems insufficient. Of note, Fer-1 is an agent only soluble in DMSO where both of the compound and the solvent could cause delay in metabolic activation, thus impeding the reliability of some reports^113,114^. On the other hand, the utility of Fer-1 is limited due to its instability in vivo to some extent.

Third, strategy targeting a specific liver cell type using dedicated vectors seems highly desirable (hepatocyte versus HSC), since ferroptosis possesses a dual role in disparate pathogenic conditions (acute liver injury versus fibrosis). It is tempting to develop disease context-dependent therapeutic regimen for limiting side effects. What also concerns us is how to modify ferroptotic process and perhaps subsequent necroinflammatory events or adaptive immune response across pathologies^115^.

Finally, it should be acknowledged that numerous precedents do exist regarding the failure of “antioxidants” treatment in clinical trials. As a matter of fact, not all “antioxidants” are effective by targeting lipid peroxidation, thus little success so far in the development of such compounds specifically in the context of disease prevention^116^. We are supposed to keep eyes on whether documented ferroptosis inducers/inhibitors fare in clinical setting. Moreover, recent studies uncovered the overexpression of apoptosis-inducing factor mitochondrial-2 (AIFM2) fully complement GPx4 depletion^117,118^. AIFM2 has been hereafter renamed ferroptosis suppressor protein-1 (FSP1) due to its critical role in a second FSP1–Q10–NADPH system, independent of the canonical GSH-based GPx4 pathway, which may regulate ferroptosis execution. Collectively, above findings spark a plethora of targets to be exploited, either standalone or in combination.

Although obstacles do exist in the way of future investigation, we still believe that sophisticate manipulation of ferroptosis has promising and essentially untapped therapeutic potential. Inhibition of ferroptosis can be utilized in the prevention and/or protection against various liver injury due to oxidative stress, lipotoxicity as well as immunogenic intermediaries, whereas induction of ferroptosis can be applicable in the treatment of challenging and standard therapy-resistant malignancies^119^. The sparkle on modification of ferroptosis is that this modality (likely) only operates when pathogenic conditions primarily drive tissue dysfunction, whereas many other types of RCD are secondary to necrotic-inflammatory processes. Furthermore, it has been longstanding recognition that iron accumulation and lipid peroxidation contribute to the pathogenesis of a myriad of diseases. At last, the solid fundamental established and ever-growing scope in relevant research will accelerate discoveries of chemical probes/regulators monitoring ferroptotic pathway, and wide evaluation of ferroptosis-modulating approaches in clinical trials. In a word, some hype but increasing hope could be anticipated by the population of cell death researchers and clinicians.
